# Physical Exercise and Psychological Distress: The Mediating Roles of Problematic Mobile Phone Use and Learning Burnout among Adolescents

**DOI:** 10.3390/ijerph18179261

**Published:** 2021-09-02

**Authors:** Yansong Li, Qilong Sun, Mingzhe Sun, Peishuai Sun, Qihui Sun, Xue Xia

**Affiliations:** 1 School of Kinesiology, Shanghai University of Sport, Shanghai 200438, China; li.yansong_@outlook.com; 2 Liaocheng Infant Normal School, Liaocheng 252699, China; sql.lns@outlook.com (Q.S.); smz.lns@outlook.com (M.S.); sps.lns@outlook.com (P.S.); sqh.lns@outlook.com (Q.S.); 3 School of Psychology, Shanghai University of Sport, Shanghai 200438, China

**Keywords:** physical exercise, problematic mobile phone use, learning burnout, psychological distress, adolescent

## Abstract

Psychological distress among adolescents adversely affects their development and negatively impacts them later in life. The aim of the present study was to determine whether an association exists between physical exercise and psychological distress and to explore the roles of problematic mobile phone use and learning burnout with respect to this association. A total of 2077 Chinese adolescents were evaluated by using the Physical Exercise Questionnaire, the Self-rating Questionnaire for Adolescent Problematic Mobile Phone Use, the Learning Burnout Questionnaire, and the Depression Anxiety Stress Scale-21. A serial multiple mediation model was constructed using the SPSS PROCESS macro. The results showed that physical exercise was negatively associated with psychological distress in this Chinese adolescent population. Serial multiple mediation analysis revealed that problematic mobile phone use and learning burnout both independently and serially mediated the association between physical exercise and psychological distress. These findings provide evidence suggesting that increased attention should be given to problematic mobile phone use and learning burnout when establishing and implementing specific strategies that leverage greater participation in physical exercise to decrease psychological distress in adolescents.

## 1. Introduction

Adolescence is a period of transition from childhood to adulthood, a crucial stage in life in which health-related behaviors and conditions typically start or are reinforced. It is also a period characterized by marked physical and psychosocial changes [1]. Although most adolescents successfully navigate this period, a substantial minority experience distress [2]. Psychological distress is defined as a state of emotional suffering characterized by depression and anxiety symptoms that are difficult to differentiate and may be accompanied by somatic symptoms [3]. Increasing levels of psychological distress increase the likelihood that the person will meet the diagnostic criteria of a mental disorder [4]. Psychological distress is more prevalent among adolescents than in the general population and may contribute to lower educational achievement and reduced overall health, both of which substantially impact them in later adulthood [3]. A non-negligible proportion of adolescents in many countries experience psychological distress. One study found that 32.9% of adolescents across four countries in Asia have experienced psychological distress [5]. In a school-based sample of Canadian adolescents, 35.1% experienced psychological distress, a percentage significantly higher than any other sector of the Canadian population [6]. Furthermore, 20.6% of adolescents in Tanzania [7], 20.8% of adolescents in India [8], and 23.3% of adolescents in Morocco [9] reported experiencing psychological distress. Given the high rates of adolescent psychological distress and the negative health impacts later in adulthood, it is crucial that the associated risk factors are identified and strategies are determined to ameliorate this condition.

The present study investigating psychological distress in adolescents is based on Salmon’s unifying theory and on the temporal self-regulation theory for physical activity [10,11]. Salmon’s unifying theory suggests that exercise may initiate numerous beneficial processes. For example, exerting the body may lead to physiological benefits, while engaging in exercise with others or self-mastery of a sport contributes to psychological well-being [11]. Exertion may also alleviate anxiety and depression as well as increase resistance to the physiological and emotional consequences of psychological stressors [11]. Physical activity may enhance the executive functions controlling behavior, thoughts, and emotions, which may in turn increase exercise participation [12,13]. According to the temporal self-regulation theory for physical activity, executive function has a conceptual role in the self-regulatory processes that are involved in an individual continuing to participate in physical activity over time. Repeatedly engaging in physical activity then tends to promote other positive health behavioral changes [10].

Increasing evidence suggests that exercise, either alone or in conjunction with other treatments, benefits numerous mental health concerns [14,15]. For example, in the Boehm and Kubzansky model, exercise is classified as a restorative behavior in relation to psychological distress [16,17]. Some cross-sectional studies have suggested an association between physical exercise and psychological distress in adults [18,19]. A population-based study conducted across 13 years also showed that both higher levels of light and moderate-to-vigorous physical activity protect against future psychological distress [20]. However, few studies have examined how physical activity is related to psychological distress in adolescents [21,22]. Moreover, adolescents tend to be physically inactive with increasing age [23], with the most marked decrease in physical activity occurring between the ages of 15 and 16 years, around the transition from junior to senior high school [24]. Conversely, prevalence rates of psychological distress, such as depression and anxiety, increase with age, especially from the mid-teens (14–16 years) [25]. Considering the marked changes in physical exercise and psychological distress during adolescence, a better understanding of the association between the two is important to offer adolescents evidence-based choices of healthy lifestyle behaviors to not only deal with potential psychological problems during adolescence but also to apply throughout their lives.

The widespread use of mobile phones has been associated with physical inactivity worldwide [26]. In China, 98.6% of 817 million people access the internet using a mobile phone, and 18% of these individuals are adolescents, who use mobile phones as their most common platform to access the internet [27]. Although mobile phones are used for study and work as well as for social activities, their excessive use is associated with potentially negative consequences. Problematic mobile phone use is defined as excessive use, which is associated with addiction-like symptoms, including craving, salience, tolerance, dependence, and loss of control, and may lead to lower academic achievement, more emotional problems, and compromised physical or psychological health [28,29]. A systematic review found that the prevalence of problematic mobile phone use among adolescents increased from 6.3% in 2011 to 16% in 2016 [30]. Evidence from recent studies supports problematic mobile phone use as a risk factor for reduced mental health among adolescents, finding a significant association between problematic mobile phone use and the severity of either depression or anxiety [31,32,33]. In addition, adolescents often display both physical inactivity and problematic mobile phone use, with previous studies indicating that either alone is associated with poorer mental health [26]. That adolescents often show both behaviors indicates that the development of specific interventions to decrease psychological distress is warranted.

Problematic mobile phone use among adolescents leads to inadequate study time and a consequent inability to maintain adequate academic progress, which may eventually lead to learning burnout [34]. Learning burnout refers specifically to academics and is characterized by a combination of emotional exhaustion, cynicism, and low effectiveness [35,36]. Identifying approaches to alleviate learning burnout is important owing to its prevalence among adolescents and its associated negative sequela, including not only a decreased desire to learn [37] but also low academic performance [38], and an increased potential for dropping out of school [39]. Burnout is also associated with both depression and anxiety [40]. A systematic review and meta-analysis by Koutsimani et al. [41] found that burnout and depression as well as burnout and anxiety are independent and robust constructs that are interconnected, share some common characteristics, and may develop in tandem. Maske et al. [42] reported that in a population of individuals diagnosed as having burnout, 59% were also diagnosed as having an anxiety disorder, 58% as also having an affective disorder (i.e., depression or a depressive episode), and 27% as also having a somatoform disorder. Given the negative behavioral and psychological consequences, it is important to see how modifiable health or risk behaviors may affect burnout. One study has shown that among the numerous lifestyle and health-behavior factors assessed, physical activity was most closely associated with burnout [43]. Consistent with this finding, Tang et al. [44] stated in their recent systematic review that exercise is likely the most effective of all interventions for learning burnout. Although the beneficial effects of exercise on burnout are compelling, the association between exercise and learning burnout has been less studied in adolescents.

During adolescence, numerous complex physical and psychological transformations take place and many behaviors (both health- and risk-related) are formed. Although the clustering or combining of these behaviors may have additive effects on mental health outcomes, to our knowledge, no study has explored the joint role of problematic mobile phone use and learning burnout as mediators of the association between physical exercise and psychological distress. Thus, the present study examined the direct effects of physical exercise on psychological distress in adolescents and also assessed whether any detected association of physical exercise with psychological distress was mediated by problematic mobile phone use or learning burnout. We specifically tested the following four hypotheses: (1) physical exercise has a direct effect on psychological distress; (2) physical exercise has an indirect effect on psychological distress through the mediator of problematic mobile phone use; (3) physical exercise has an indirect effect on psychological distress through the mediator of learning burnout; and (4) physical exercise has an indirect effect on psychological distress through sequential mediation of problematic mobile phone use and learning burnout.

## 2. Materials and Methods

### 2.1. Participants

We conducted this cross-sectional study from December 2020 to February 2021 as part of a broader study of physical exercise and mental health among adolescents in Shandong province, China. We recruited 2407 adolescents through the school’s intranet system and selected participants who answered the study questionnaires by using a mobile phone app. The inclusion criteria were being between 10 and 19 years of age and having unrestricted use of a mobile phone. The exclusion criteria were finishing the questionnaire in less than 2 min or responding inaccurately to an instructed item (e.g., not answering “strongly agree” to an item stating: respond with “strongly agree” for this item). Valid responses were obtained from 2077 participants (86.3%).

The study was conducted in accordance with the recommendations of the World Medical Association’s Declaration of Helsinki of 1975 and was approved by the Shanghai University of Sport Ethics Committee. Written informed consent was obtained from all participants and their parents or guardians.

### 2.2. Measures

#### 2.2.1. Sociodemographic Variables

Sociodemographic information, including age, sex, and residential area, was obtained from the participants. Socioeconomic status was also identified based on the family income as perceived by the participants and was rated on a three-point scale from 1 (low) to 3 (high) [45].

#### 2.2.2. Physical Exercise

Physical exercise was assessed via the Physical Exercise Questionnaire, a widely used evaluation that has shown high reliability and validity for Chinese adolescents [46]. The questionnaire includes eight items that are separated into two categories: exercise commitment and exercise persistence. Participants were asked to rate themselves on questions using a 5-point Likert scale ranging from 1 (strongly disagree) to 5 (strongly agree). The total score indicates the physical exercise level of the participants. Cronbach’s alpha for this assessment in the present study was 0.863, which signifies good internal consistency. Cronbach’s alpha was 0.777 for exercise commitment and 0.820 for exercise persistence, both indicating adequate internal consistency.

#### 2.2.3. Problematic Mobile Phone Use

Problematic mobile phone use was measured using the Self-rating Questionnaire for Adolescent Problematic Mobile Phone Use [47]. There were 13 items within three content subscales—withdrawal symptoms, craving, and physical and mental health status. Participants responded using a 5-point Likert scale, from 1 (never) to 5 (always). The scores ranged from 13 to 65, and participants with scores ≥29 were considered problematic mobile phone users. In the present study, the Cronbach’s alpha coefficient for this assessment was 0.926, which represents high reliability. Cronbach’s alpha coefficients for the subscales of withdrawal symptoms, craving, and physical and mental health statuses ranged from 0.693 to 0.872, signifying adequate to sound internal consistencies.

#### 2.2.4. Learning Burnout

Learning burnout data were collected using the Learning Burnout Questionnaire [48], which includes 20 items that are separated into three categories (factors): low personal accomplishment, dejection, and improper behavior. Participants were asked to rate themselves on questions using a 5-point Likert scale ranging from 1 (did not apply to me at all) to 5 (applied to me totally), with a total score ranging from 20 to 100. All factor scores were summed to calculate the total score, and higher total scores indicated higher levels of learning burnout, which were categorized as low (<33), average (33–66), and high (>66). Cronbach’s alpha for this assessment in our sample was 0.864, showing good reliability. Cronbach’s alpha coefficients for low personal accomplishment, dejection, and improper behavior were 0.738, 0.870, and 0.663, respectively, which were all within acceptable parameters.

#### 2.2.5. Psychological Distress

Psychological distress was obtained using the Depression Anxiety Stress Scale-21 (DASS-21), a 21-item self-report scale providing an assessment of the severity of psychological distress as a total score and subscores in three domains: symptoms of depression, anxiety, and stress [49]. Participants rated the extent to which certain experiences applied to them during the previous week on a 4-point Likert scale ranging from 0 (did not apply to me at all) to 3 (applied to me very much or most of the time). The sum of each subscale provided an indicator of general psychological distress. Scoring 25 and above denotes the presence of distress, with higher scores indicating higher levels of psychological distress. Cronbach’s alpha for this assessment in the present study was 0.949, and Cronbach’s alpha coefficients for the psychological distress subscales were 0.877 (depression), 0.864 (anxiety), and 0.862 (stress). Previous studies have provided evidence for the validity and reliability of the DASS-21 as a measure of psychological distress in Chinese adolescents [50].

### 2.3. Statistical Analysis

Descriptive characteristics of the participants are presented as means ± standard deviation (SDs) or as percentages. Age, sex, and socioeconomic status were selected as covariates since they were associated with the main variables. Partial correlation coefficients were estimated to examine the associations among physical exercise, problematic mobile phone use, learning burnout, and psychological distress while controlling for age, sex, and socioeconomic status. Harman’s single-factor test was used to test for common method bias. Serial multiple mediation analysis was conducted to examine whether the association of physical exercise with psychological distress was mediated by problematic mobile phone use or by learning burnout using Hayes’ PROCESS macro for SPSS (Model 6) [51]. Age, sex, and socio-economic status were adjusted in this model. Mediation hypotheses were tested with bootstrapping using resampling of 5000 samples to calculate 95% confidence intervals (CIs). If the 95% CI did not contain zero and the *p* value was < 0.05, the results were deemed statistically significant. Statistical analyses were performed using SPSS version 22.0 (IBM Corp., Armonk, NY, USA).

## 3. Results

### 3.1. Descriptive Statistics

The recruited sample comprised 2407 adolescents and reflected the demographic composition of the school population. Valid responses were obtained from 2077 participants, with a mean (SD) age of 16.27 (1.02) years, and 1797 [86.5%] were girls (Table 1). In this population of adolescents, the mean (SD) physical exercise score was 27.82 ± 4.81. More than one-third of participants (37.0%) reported problematic mobile phone use. Learning burnout scores were categorized as low for 2.5% of the population, average for 84.1%, and high for 13.4%. The estimated prevalence of psychological distress in the population was 43.7%.

### 3.2. Partial Correlation Analysis

Partial correlations among physical exercise, problematic mobile phone use, learning burnout, and psychological distress while controlling for age, sex, and socioeconomic status are presented in Table 2. All the following partial correlation coefficients were statistically significant, with *p* < 0.001 for each correlation. Physical exercise was negatively correlated with problematic mobile phone use (*r* = −0.235), learning burnout (*r* = −0.391), and psychological distress (*r* = −0.263). Problematic mobile phone use was positively correlated with learning burnout (*r* = 0.503) and psychological distress (*r* = 0.435). A positive correlation was also found between learning burnout and psychological distress (*r* = 0.486).

### 3.3. Serial Multiple Mediation Analysis

**Figure 1:**
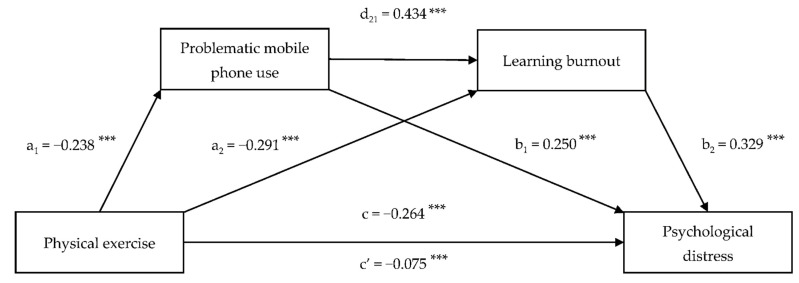
Serial multiple mediation model showing the association between physical exercise and psychological distress with problematic mobile phone use and learning burnout as mediators, adjusted for age, sex, and socioeconomic status. Standardized regression coefficients are presented. *** *p* < 0.001.

Before conducting the serial multiple mediation analysis, a common method bias test was examined. The variance of the most important factor was 26.4%, less than the critical value (i.e., 40%) determined by Harman’s single-factor test, which indicated that no serious common method bias existed in the present study. The results of the serial multiple mediation analysis are presented in Table 3 and Figure 1. Physical exercise was negatively associated with problematic mobile phone use (a_1_ = −0.238, *p* < 0.001) and learning burnout (a_2_ = −0.291, *p* < 0.001). Problematic mobile phone use was positively associated with learning burnout (d_21_ = 0.434, *p* < 0.001) and psychological distress (b_1_ = 0.250, *p* < 0.001). Learning burnout was positively associated with psychological distress (b_2_ = 0.329, *p* < 0.001). The association of physical exercise with psychological distress (c = −0.264, *p* < 0.001) was partially mediated by problematic mobile phone use and learning burnout (c–c’ = −0.189, *p* < 0.001).

Following mediation hypotheses testing via bootstrapping, we determined the effect of serial multiple mediation on the association between physical exercise and psychological distress (Table 4). The percentage of total IEs mediated by problematic mobile phone use and learning burnout was 71.6%. The total IEs included three significant pathways: (1) IE_1_: physical exercise–problematic mobile phone use–psychological distress (a_1_ × b_1_ = −0.059; 95% CI, −0.077 to −0.044); (2) IE_2_: physical exercise–learning burnout–psychological distress (a_2_ × b_2_ = −0.096; 95% CI, −0.115 to −0.077); and (3) IE_3_: physical exercise–problematic mobile phone use–learning burnout–psychological distress (a_1_ × d_21_ × b_2_ = −0.034, 95% CI, −0.042 to −0.026). IE_1_, IE_2_, and IE_3_ accounted for 22.3%, 36.4%, and 12.9% of the total effect, respectively.

## 4. Discussion

The present study is, to our knowledge, the first to analyze a four-way association of physical exercise, problematic mobile phone use, learning burnout, and psychological distress in adolescents by using serial multiple mediation analysis. Our results supported our four study hypotheses. These findings contribute to current knowledge by providing evidence indicating that physical exercise plays a crucial role in psychological distress among adolescents and by suggesting that physical exercise may be related to reduced problematic mobile phone use, decreased learning burnout, or both, which could affect psychological distress in adolescents.

That 43.7% of our population of adolescents experienced psychological distress reaffirms the high prevalence of psychological distress among adolescents and suggests that the need to identify factors that contribute to, prevent, or alleviate psychological distress during adolescence is warranted. Consistent with the literature, our data showed that there was a close association of physical exercise with psychological distress in adolescents, with a direct effect accounting for 28.4% of the association. Although the potential biological mechanisms underlying this direct effect are uncertain, several mechanisms may be plausible. For example, exercise may lead to greater diversity in the microbiome of in individual along with increasing genera of the phylum Firmicutes, which may be linked to decreased psychological distress [52]. Physical inactivity may also interfere with the hypothalamic–pituitary–adrenal axis and alter serum cortisol levels to cause psychological distress [53]. Exercise has been shown to enhance endorphin secretion in the brain, and according to the “endorphin hypothesis,” mood states are dependent on brain endorphin levels. Enhanced endorphin release may also decrease pain or lead to euphoria, both of which may reduce depression or anxiety symptoms [26,54]. The mechanisms that may explain the association between physical fitness and mental health may also apply to physical activity and mental health, with physical fitness defined as the ability to perform a physical activity or to perform physical exercise using most of the body structures. [55,56]. Indeed, a recent study showed that both “insufficient” physical activity and “lower” levels of physical fitness are associated with “high” psychological distress [57]. Findings in a study by Rodriguez-Ayllon et al. [58] suggest that greater muscular strength may have a positive influence on psychological distress. Our previous work indicated that agility moderates the association between physical fitness and anxiety [59]. These results suggest that exercise interventions based on physical fitness may provide valuable information to inform strategies to ameliorate psychological distress in adolescents. Thus, future studies could take physical fitness into account to offer a new perspective for furthering our understanding of how physical exercise affects psychological distress.

Our results showed that the indirect mediating effects of problematic mobile phone use and of learning burnout on the association of physical exercise with psychological distress in adolescents accounted for 71.6% of the total effect. Of the three significant pathways, problematic mobile phone use (IE_1_) and learning burnout (IE_2_) played indirect roles as independent mediating variables in the association of physical exercise with psychological distress in adolescents. Consistent with recent studies, our results found a close association between physical exercise and problematic mobile phone use. Actively engaging in physical exercise may contribute to decreasing both screen time and sedentary behavior among adolescents so that they have less time to devote to mobile phone use and a lower chance of becoming addicted to it [60]. From a neurophysiological perspective, both exercise and mobile phone use may activate similar pathways in the brain. Exercise may attenuate problematic mobile phone use via its effects on key brain structures involved in reward and inhibitory control [61]. Excessive mobile phone use may also create isolation and loneliness, and adolescents may experience symptoms associated with depression, anxiety, and stress [62]. Xie et al. [26] found that joint insufficient physical exercise and problematic mobile phone use were significantly associated with increased depression symptoms. These results together with our findings for IE_1_ suggest that physical exercise may have a positive impact by decreasing the overuse of mobile phones, making adolescents less likely to become problematic mobile phone users and protecting them from the associated psychological distress [63]. Thus, along with increasing physical exercise, decreasing mobile phone use may prevent or alleviate psychological distress.

The mediating effect of learning burnout on the association of physical exercise with psychological distress in adolescents, IE_2_, accounted for the greatest portion of the total effect (36.4%), suggesting that learning burnout may, to some extent, underlie the degree of this association. Physical exercise is a personal resource that an individual may use to reduce stress and prevent burnout symptoms [64]. It temporarily releases individuals from stress, allowing for the renewal of other personal resources to confront demands [65,66]. Given that exercise increases heart rate, blood pressure, and central nervous system neurotransmitter availability, it may also prevent learning burnout by decreasing an individual’s physical vulnerability to stress [67,68,69]. Burnout syndrome has been associated with mental health problems, including depression, anxiety, and stress [40], constructs that may be similar on a biological level [41]. Kasemy et al. [70] found that emotional exhaustion is associated with some proinflammatory markers, including interleukin 6, TNFα, and coenzyme Q10, and inflammation is a key factor in the development of some mental health problems [71], including depression [72] and anxiety [73]. Our findings are consistent with the importance of learning burnout on psychological distress and suggest that learning burnout plays a crucial role in the association of physical exercise with psychological distress in adolescents.

The serial multiple mediation model used in our analysis offers a more granular understanding of the pathways that associate physical exercise with psychological distress: the association was partially mediated by problematic mobile phone use and learning burnout. The IE_3_ pathway indicated that physical exercise was sequentially correlated with problematic mobile phone use in the first step and further affected the onset of learning burnout, which was associated with the risk of psychological distress. Researchers are increasingly focusing on the association between problematic mobile phone use and learning burnout [34,74]. Our results supported such a positive association, that is, increase in the former was associated with increase in the latter. One study finds that individuals who are highly addicted to social media have lower mindfulness and may cope with stress by using emotion-based mechanisms, an approach that increases emotional exhaustion and thus burnout [75]. Problematic mobile phone use may also lead to a variety of health problems that consume energy, taking away from learning resources and ultimately leading to burnout in adolescents [34]. Furthermore, Salmela-Aro et al. [76] suggested that addictive behavioral patterns as a potential risk factor for learning burnout may contribute to depressive symptoms in adolescents. Therefore, factors of both problematic mobile phone use and learning burnout should be considered when designing strategies aimed at alleviating psychological distress through physical exercise.

Our findings have important theoretical and practical implications for understand-ing the prevention and alleviation of psychological distress. On a theoretical level, our se-rial mediation model provides strong supporting evidence for Salmon’s unifying theory and the temporal self-regulation theory for physical activity and offers an important addi-tion to these theories. Our findings not only confirm the association between physical ex-ercise and psychological distress but also further clarify the mechanisms underlying how physical fitness exerts this effect on adolescents’ psychological distress. These findings may prove useful for future research studying the causal association between health- and risk-related behaviors and psychological health. In terms of practical implications, the re-sults from our model suggested that physical exercise, problematic mobile phone use, and learning burnout were associated, directly or indirectly, with psychological distress in ad-olescents. This means that in addition to considering a direct link between physical exer-cise and psychological distress, attention should also be given to problematic mobile phone use and learning burnout. Our findings provide preliminary data that incorporat-ing methods to decrease problematic mobile phone use and learning burnout may lead to better outcomes when designing physical exercise programs to improve psychological distress in adolescents.

Some study limitations should be considered when interpreting our results. This study used a cross-sectional design, which cannot provide evidence for causality. Although participant information was collected using standardized questionnaires, the data were self-reported. Data collected using self-report and questionnaires may be susceptible to memory bias and measurement errors, and participants’ answers may be influenced by social expectations. More objective methods of data collection and measurements, such as the use of accelerometers and mobile monitoring software to assess physical exercise, should be considered in future research.

## 5. Conclusions

Our serial multiple mediation model informs the associations among physical exercise, problematic mobile phone use, learning burnout, and psychological distress. Physical exercise may be an effective strategy for preventing or ameliorating psychological distress in adolescents. Problematic mobile phone use and learning burnout both independently and serially mediated the association between physical exercise and psychological distress. These findings may contribute to the better understanding of potential antecedents of psychological distress among adolescents, offering an integrated view and informing the development of effective intervention programs. The findings suggest that both problematic mobile phone use and learning burnout should be considered when developing specific strategies that leverage greater participation in physical exercise to decrease psychological distress in adolescents.

## Figures and Tables

**Table 1:** Demographic characteristics of participants.

Characteristic	No. (%) of 2077 Participants
Age, mean ± SD, y	16.27 ± 1.02
Sex	
* male*	280 (13.5)
* female*	1797 (86.5)
Residential area	
* rural*	731 (35.2)
* urban*	1346 (64.8)
Perceived family income	
* low*	212 (10.2)
* medium*	1842 (88.7)
* high*	23 (1.1)

**Table 2:** Partial correlation analysis results for each variable.

Variable	Test Score (mean ± SD)	Correlation Coefficient (*r*)			
		1	2	3	4
1. Physical exercise	27.82 ± 4.81	-			
2. Problematic mobile phone use	26.10 ± 9.45	−0.235 ***	-		
3. Learning burnout	56.04 ± 11.09	−0.391 ***	0.503 ***	-	
4. Psychological distress	24.54 ± 21.52	−0.263 ***	0.435 ***	0.486 ***	-

Note: *** *p* < 0.001.

**Table 3:** Direct effects of variables in the serial multiple mediation model.

Paths	*β*	SE	*t*	LLCI	ULCI
Physical exercise–Problematic mobile phone use	−0.238	0.022	−11.022 ***	−0.280	−0.195
Physical exercise–Learning burnout	−0.291	0.019	−15.618 ***	−0.327	−0.254
Problematic mobile phone use–Learning burnout	0.434	0.018	23.560 ***	0.398	0.470
Physical exercise–Psychological distress	−0.075	0.020	−3.696 ***	−0.114	−0.035
Problematic mobile phone use–Psychological distress	0.250	0.021	11.732 ***	0.208	0.292
Learning burnout–Psychological distress	0.329	0.023	14.570 ***	0.285	0.373

Note: *β* = standardized regression coefficient; SE = standard error; LLCI = lower limit of the confidence interval; ULCI = upper limit of the confidence interval. *** *p* < 0.001.

**Table 4:** Indirect effects of physical exercise on psychological distress in adolescents.

Indirect Effect	*β*	Bootstrap SE	95% CI	Percentage Accounting for Total Effect
Total IEs	−0.189	0.013	−0.216 to −0.164	71.6
IE_1_	−0.059	0.008	−0.077 to −0.044	22.3
IE_2_	−0.096	0.010	−0.115 to −0.077	36.4
IE_3_	−0.034	0.004	−0.042 to −0.026	12.9

Note: β = standardized regression coefficient; SE = standard error; CI = confidence interval; IE = indirect effect; IE_1_ = physical exercise–problematic mobile phone use–psychological distress; IE_2_ = physical exercise–learning burnout –psychological distress; IE_3_ = physical exercise–problematic mobile phone use–learning burnout–psychological distress. The IE is statistically significant at the 95% CI when the CI does not include zero.

## Data Availability

The data that support the findings of this study are available from the corresponding author, upon reasonable request.
